# Beneficial Chromosomal Integration of the Genes for CTX-M Extended-Spectrum β-Lactamase in *Klebsiella pneumoniae* for Stable Propagation

**DOI:** 10.1128/mSystems.00459-20

**Published:** 2020-09-29

**Authors:** Eun-Jeong Yoon, Bareum Gwon, Changseung Liu, Dokyun Kim, Dongju Won, Sung Gyun Park, Jong Rak Choi, Seok Hoon Jeong

**Affiliations:** a Department of Laboratory Medicine, Yonsei University College of Medicine, Seoul, South Korea; b Research Institute of Bacterial Resistance, Yonsei University College of Medicine, Seoul, South Korea; University of Illinois at Chicago

**Keywords:** CTX-M, extended-spectrum β-lactamases, *Klebsiella pneumoniae*, chromosomal integration, IS*Ecp1*, IS*26*

## Abstract

The acquired CTX-M-type extended-spectrum-β-lactamase (ESBL)-producing *Enterobacterales* are of great concern in clinical settings because they limit therapeutic options for patients infected by the pathogens. An intriguing clonality of CTX-M ESBL-producing Klebsiella pneumoniae blood isolates was observed from a national cohort study, and comparative genomics were assessed for the 115 K. pneumoniae blood isolates carrying the *bla*_CTX-M_ gene. The plasmid preference of particular clones of a sequence type (ST) was assessed by liquid mating. A quarter of the *bla*_CTX-M_ gene-carrying K. pneumoniae blood isolates harbor the gene in their chromosome, and most of those with the built-in *bla*_CTX-M_ gene belonged either to ST307 or ST48. Notably, all 16 K. pneumoniae ST48 isolates harbored two copies of the *bla*_CTX-M-15_ gene in the chromosome. The chromosomal integration of the *bla*_CTX-M-15_ gene was mostly derived from the IS*Ecp1*-targeting 5-bp AT-rich locus in the chromosome. The IS*26*-mediated chromosomal integration occurred when the upstream IS*Ecp1* from the *bla*_CTX-M_ gene was truncated, targeting the anchor IS*26* copy in the chromosome. Higher transfer efficiency of the *bla*_CTX-M-15_ gene-carrying FIA:R plasmid was observed in ST17 than that of the *bla*_CTX-M-14_ gene-carrying FIB:FII plasmid. The transfer efficiency of the plasmid differed by isolate among the ST307 members. The K. pneumoniae clones ST307 and ST48 harboring the *bla*_CTX-M-15_ gene in the chromosome were able to disseminate stably in clinical settings regardless of the environmental pressure, and the current population of K. pneumoniae blood isolates was constructed. Further follow-up is needed for the epidemiology of this antimicrobial resistance.

**IMPORTANCE** Dominant F-type plasmids harboring the gene have been pointed out to be responsible for the dissemination of the CTX-M extended-spectrum-β-lactamase (ESBL)-producing K. pneumoniae. Recently, the emergence of K. pneumoniae isolates with the *bla*_CTX-M_ gene in their chromosomes has been reported occasionally worldwide. Such a chromosomal location of the resistance gene could be beneficial for stable propagation, as was the Acinetobacter baumannii ST191 harboring chromosomal *bla*_OXA-23_ that is endemic to South Korea. Through the present study, particular clones were identified as having built-in resistance genes in their chromosomes, and the chromosomal integration events were tracked by assessing their genomes. The cefotaxime-resistant K. pneumoniae clones of this study were particularized as results of the fastidiousness for plasmids to acquire the *bla*_CTX-M_ gene for securing the diversity and of the chromosomal addiction of the *bla*_CTX-M_ gene for ensuring propagation.

## Abstract (precis)

Dominant F-type plasmids harboring the gene have been pointed out to be responsible for the dissemination of the CTX-M extended-spectrum-β-lactamase (ESBL)-producing K. pneumoniae. Recently, the emergence of K. pneumoniae isolates with the *bla*_CTX-M_ gene in their chromosomes has been reported occasionally worldwide. Such a chromosomal location of the resistance gene could be beneficial for stable propagation, as was the Acinetobacter baumannii ST191 harboring chromosomal *bla*_OXA-23_ that is endemic to South Korea. Through the present study, particular clones were identified as having built-in resistance genes in their chromosomes, and the chromosomal integration events were tracked by assessing their genomes. The cefotaxime-resistant K. pneumoniae clones of this study were particularized as results of the fastidiousness for plasmids to acquire the *bla*_CTX-M_ gene for securing the diversity and of the chromosomal addiction of the *bla*_CTX-M_ gene for ensuring propagation.

## INTRODUCTION

The acquired CTX-M-type extended-spectrum β-lactamases (ESBLs) belonging to the class A β-lactamases are grouped into five groups, 1, 2, 8, 9, and 25, by amino acid sequence similarity. Members within the same group share >94% identity and the members belonging to distinct groups share ≤90% identity (1). The five groups had been shown to originate from an intrinsic β-lactamase gene of different species of *Kluyvera*, i.e., both groups 1 and 2 from Kluyvera ascorbata (2, 3), groups 8 and 9 from Kluyvera georgiana (4, 5), and group 25, which remains to be identified but probably is from another member of *Kluyvera*. The insertion sequences (ISs), mostly IS*Ecp1* and, less frequently, IS*CR1*, hijacked *bla*_CTX-M_ from the chromosome of the progenitor *Kluyvera* spp. and recruited it into a plasmid, which is then transferred to clinically relevant enterobacterial isolates. The ISs are recognized upstream of the *bla*_CTX-M_ gene and provide portable promoter sequences stronger than the natural promoter sequences (6).

After the emergence of CTX-M ESBL-producing Escherichia coli in clinical settings in 1989 (7), CTX-M ESBL-producing *Enterobacterales* spread rapidly in the world, and their high prevalence is a grave concern in clinical settings, as the treatment options for patients infected by these pathogens are limited (8, 9). Dominance of the *bla*_CTX-M-14_ and *bla*_CTX-M-15_ genes in *Enterobacterales* clinical strains is achieved by their association with the mobile transposition element IS*Ecp1* and its location in prevalent F-type plasmids (10, 11). Such conjugative plasmids, often carrying antimicrobial resistance genes, maintain their occupancy in bacterial populations by using plasmid addiction systems, such as the toxin-antitoxin (TA) systems that kill the plasmid-free daughter cell through a stable toxin and an unstable antitoxin (12). In addition, clonal expansion of a disseminated bacterial clone supports the successful spread of CTX-M ESBL-producing *Enterobacterales.* In contrast to the obvious dominance of the E. coli clone sequence type 131 (ST131) among the CTX-M ESBL producers (8), the *bla*_CTX-M_ ESBL gene-carrying Klebsiella pneumoniae is known to be devoid of clonality (13, 14). The emergence of K. pneumoniae isolates carrying the *bla*_CTX-M_ gene in their chromosomes, devoid of clonality, were reported rarely from the beginning of the 2010s (15–18).

**FIG 1:**
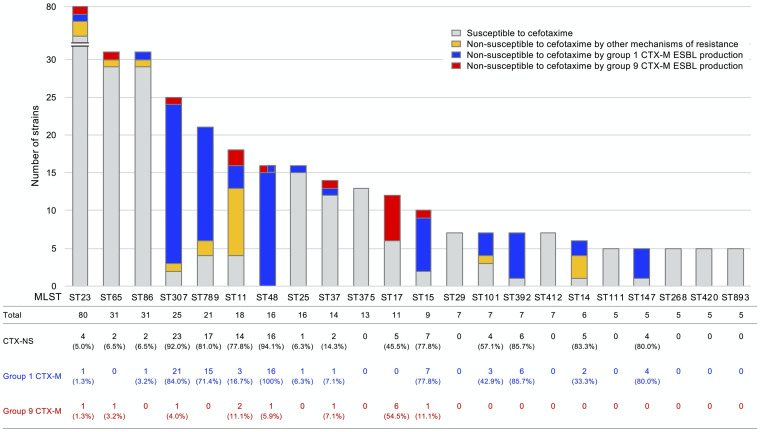
Prevalence of cefotaxime-nonsusceptible and CTX-M ESBL-positive K. pneumoniae blood isolates. The STs of more than 5 isolates were included in the graph. Bars represent the numbers of isolates of each group. Gray, susceptible to cefotaxime; yellow, nonsusceptibility to cefotaxime that is conferred by mechanisms of resistance other than CTX-M ESBLs; blue, nonsusceptibility to cefotaxime that is conferred by the production of the group 1 CTX-M ESBLs; red, those with nonsusceptibility to cefotaxime that is conferred by the production of the group 9 CTX-M ESBLs.

From national antimicrobial resistance surveillance, designed as a cohort study for entire episodes of K. pneumoniae bloodstream infections occurring in a year in six general hospitals in South Korea, we were able to collect a total of 572 K. pneumoniae blood isolates, including 164 cefotaxime-nonsusceptible isolates (19). Of the cefotaxime-nonsusceptible K. pneumoniae isolates, 81.7% (134/164) harbored the *bla*_CTX-M_ ESBL gene belonging either to group 1 or to group 9 (20). As shown in Fig. 1, certain STs, i.e., ST307, ST789, ST11, ST48, ST15, ST392, and ST14, have absolute high rates of cefotaxime resistance of over 78%. Particular STs also favor harboring the *bla*_CTX-M_ gene. For instance, the cefotaxime-nonsusceptible ST48, ST789, ST392, and ST147, together with ST307, with one exception, harbored the group 1 *bla*_CTX-M_ gene, while ST17 carried only the group 9 *bla*_CTX-M_ gene. In the case of ST11 isolates, half of the cefotaxime-nonsusceptible isolates harbored the group 1 *bla*_CTX-M_ gene, and the other half had the group 9 *bla*_CTX-M_ gene. The phenomenon could be derived from the preferential acquisition of specific plasmids carrying a particular *bla*_CTX-M_ gene or the clonal dissemination together with the expansion of the clones possessing the residential *bla*_CTX-M_ gene in the chromosome. To determine how the clonality of cefotaxime resistance was achieved, *bla*_CTX-M_ ESBL gene-carrying K. pneumoniae blood isolates from the cohort study were entirely sequenced, and a comparative analysis was carried out.

## RESULTS

### Genomes of the K. pneumoniae blood isolates.

**FIG 2:**
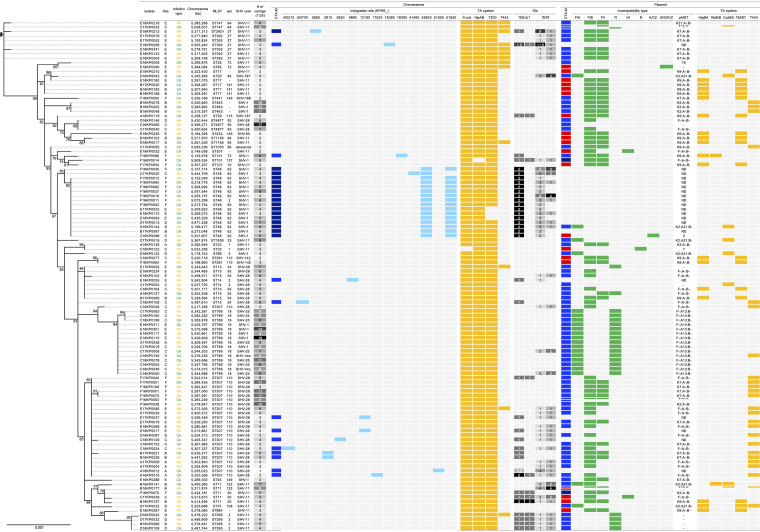
Phylogeny of the CTX-M-type ESBL-producing K. pneumoniae blood strains based on the alignment of the core genome proteins and the characteristics associated with the *bla*_CTX-M_ gene carried by each strain. Blue and red indicate the presence of the group 1 and group 9 *bla*_CTX-M_ genes, respectively. Dark blue represents the presence of two group 1 *bla*_CTX-M_ genes in the chromosome or in plasmids. Chromosomal locations of the integrated *bla*_CTX-M-15_ gene are indicated in sky blue, the presence of toxin-antitoxin (TA) systems is indicated in yellow, and the incompatibility type of the plasmid carrying the *bla*_CTX-M_ gene is indicated in green. The numbers of contigs and IS*Ecp1* and IS*26* insertion sequences are indicated as black and white heatmaps.

A total of 115 K. pneumoniae blood isolates were entirely sequenced. The contigs were numbered from 1 to 19, and the circularized chromosomes had a median size of 53,115,357 bp, ranging from 4,971,228 bp to 5,532,256 bp (Fig. 2). The ST of each isolate was determined *in silico*, and a total of 30 STs were identified. The most prevalent was ST307, which included 25 isolates. ST48 (*n* = 16), ST789 (*n* = 15), ST15 (*n* = 7), ST392 (*n* = 6) along with the *rpoB* allele of ST392-like (*n* = 1) possessing a single-nucleotide polymorphism, ST17 (*n* = 5), ST11 (*n* = 5), ST395 (*n* = 4), ST4877 (*n* = 3), and ST463 (*n* = 3) were detected. One or two isolates belonged to the other 19 different STs.

The intrinsic *bla*_SHV_ gene was extracted, and the translated sequences were used to subtype the allele. A total of 9 subtypes were identified, and one isolate lost the gene through the interruption of IS*26*. The subtypes of SHV β-lactamases 28 (*n* = 36), 11 (*n* = 34), and 1 (*n* = 28) were prevalent, and SHV-28 ESBL was identified in particular clones of ST14, ST15, ST4877, and ST307 (Fig. 2; also see Fig. S1 in the supplemental material). The alleles of intrinsic SHV had fewer than 5 mismatches among 286 amino acids, with an amino acid identity of >98.2%. The molecular phylogeny from the multiple alignments of SHV alleles was poor, and no clear correspondence to the phylogenetic tree of STs (Fig. S1) or to that of the core genome (Fig. 2) was observed.

10.1128/mSystems.00459-20.3 FIG S1 Correlation between the intrinsic SHV and sequence type. Numbers of the isolates corresponding to the intrinsic SHV and ST are indicated in the cell, and the populations are indicated in the green heatmap. Gray cells indicate no corresponding isolate. The absence of SHV was due to genetic recombination by IS*26*. The molecular phylogeny was analyzed using either the amino acid sequences of intrinsic SHV (right) or the concatenated nucleic acid sequences of the seven alleles of the ST (top). Statistical analysis of the phylogeny of each isolate was conducted through 100 bootstraps, and those over 60 are indicated at the node. Download FIG S1, TIF file, 2.4 MB. Copyright © 2020 Yoon et al. 2020 Yoon et al. This content is distributed under the terms of the Creative Commons Attribution 4.0 International license.

A total of 29 isolates harbored CTX-M-15 coding genes in their chromosome; seven of these isolates harbored an extra *bla*_CTX-M-15_ gene in a plasmid, and one ST48 isolate carried a plasmid harboring the *bla*_CTX-M-14_ gene (Fig. 2). Characteristically, the chromosomes of all 16 ST48 isolates and the ST392-like isolate had two copies of the gene. The 16 ST48 isolates with two copies of the *bla*_CTX-M-15_ gene were closely related to each other, but no single strain was supported by the core genome multilocus sequence typing (cgMLST) (Fig. S2). Two isolates belonging to ST147 and ST307 carried two plasmids harboring the *bla*_CTX-M-15_ gene, and one ST11 isolate possessed both the *bla*_CTX-M-15_ gene-carrying plasmid and the *bla*_CTX-M-14_ gene-carrying plasmid.

10.1128/mSystems.00459-20.4 FIG S2 Minimum spanning trees of the six major STs having the most numerous isolates. Isolates belonging to ST15, ST17, ST48, ST307, ST392, or ST782 are presented. Colors of the nodes are assigned by the hospital from which the isolates were recovered, and the size of each node reflects logarithmically the number of isolates. The length of node links is presented in log scale, and the values of absolute distance are indicated in red. Download FIG S2, TIF file, 2.7 MB. Copyright © 2020 Yoon et al. 2020 Yoon et al. This content is distributed under the terms of the Creative Commons Attribution 4.0 International license.

### Plasmids carrying the *bla*_CTX-M_ gene.

**FIG 3:**
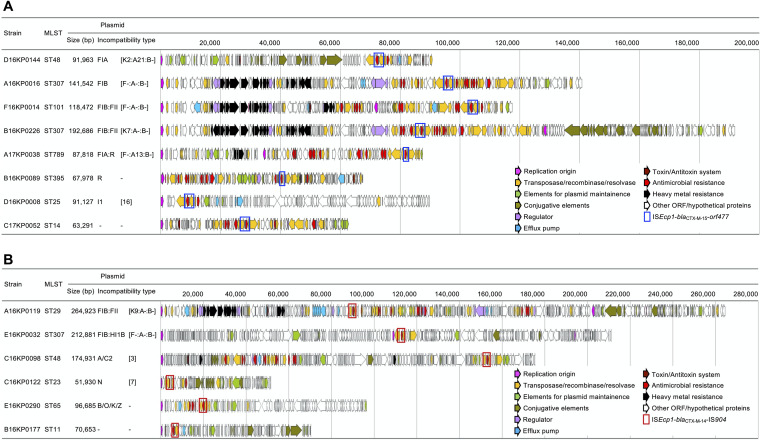
Schematic structure of the representative plasmids harboring the group 1 (A) or group 9 (B) *bla*_CTX-M_ genes.

**(i) Incompatibility types of the *bla*_CTX-M_ gene-carrying plasmids.** The characteristics and gene contents of representative plasmids are schematically presented in Fig. 3 and Table 1. Among the plasmids carrying the group 1 *bla*_CTX-M_ genes, the dominant FIB:FII-type plasmids were observed to be diverse in terms of size and genetic components. However, the mosaic plasmids of FIB and FII carried characteristic gene contents of those plasmids, including clustered antimicrobial genes bracketed by varied ISs and heavy-metal resistance gene clusters in the FIB plasmids and the *tra*-type conjugative elements in FII plasmids. The FIB:FII plasmids harboring the *bla*_CTX-M-14_ gene were observed to have more mosaicism than those carrying the *bla*_CTX-M-15_ gene, as the length was ca. 100 kb longer, and more genetic elements unrelated to FIB:FII were identified. The plasmids of infrequent incompatibility types shared only the *bla*_CTX-M_ gene and the vicinity with other plasmids. ISs were gathered near the genes for antimicrobial resistance, allowing the feasible mobility of the gene. The FIB:FII plasmids carrying the *bla*_CTX-M_ gene in ST307 and ST463 isolates possessed the GNAT-related TacTA, while those in other STs have both HigBA and STM4031 (Fig. 2).

**TABLE 1:** Completeness of IS*Ecp1* upstream from the *bla*_CTX-M_ gene

Plasmid	Complete copy of IS*Ecp1*	Truncated IS*Ecp1*
Upstream from the group 1 *bla*_CTX-M_ gene^*a*^		
Total (*n* = 78)	36 (46.2%)	42 (53.8%)
FIA [K2:A21:B-] (*n* = 5)	5	0
FIA:R [F-:A13:B-] (*n* = 15)	0	15
FIB [F-:A-:B-] (*n* = 16)	5	11
FIB:FII total (*n* = 29)	*21*	*8*
[K9:A-:B-] (*n* = 3)	3	0
[K2:A-:B-] (*n* = 8)	7	1
[K7:A-:B-] (*n* = 16)	10	6
[F-:A-:B-] (*n* = 1)	1	0
[F21:A-:B-] (*n* = 1)	0	1
FIA:FIB [K2:A21:B-] (*n* = 1)	1	0
FII [K7:A-:B-] (*n* = 1)	0	1
FII:R [F-:A-:B-] (*n* = 1)	1	0
R (*n* = 6)	1	5
I1 [16] (*n* = 1)	1	0
— (*n* = 3)	1	2
Upstream from the group 9 *bla*_CTX-M_^*b*^		
Total (*n* = 20)	18 (94.7%)	1 (5.3%)
FIB:FII [K9:A-:B-] (*n* = 14)	14	0
FIB:HI1B [K-:A-:B-] (*n* = 1)	1	0
A/C2 [3] (*n* = 1)	1	0
N [7] (*n* = 1)	1	0
B/O/K/Z (*n* = 1)	0	1
— (*n* = 1)	1	1

a Truncation of IS*Ecp1* was associated with one of the ISs, IS*6100* (four FIB:FII [K7:A-:B-] plasmids in ST392), IS*Kpn11* (three FIB [K7:A-:B-] plasmids in ST307), IS*Kpn14* (four FIB [K7:A-:B-] plasmids in two of each ST15 and ST4877 strain), and IS*26* (the rest).

b Two cases had uncirculated contigs. One *bla*_CTX-M-9_ gene carried by the FIB:HI1B [K-:A-:B-] plasmids was associated with IS*CR1*.

**FIG 4:**
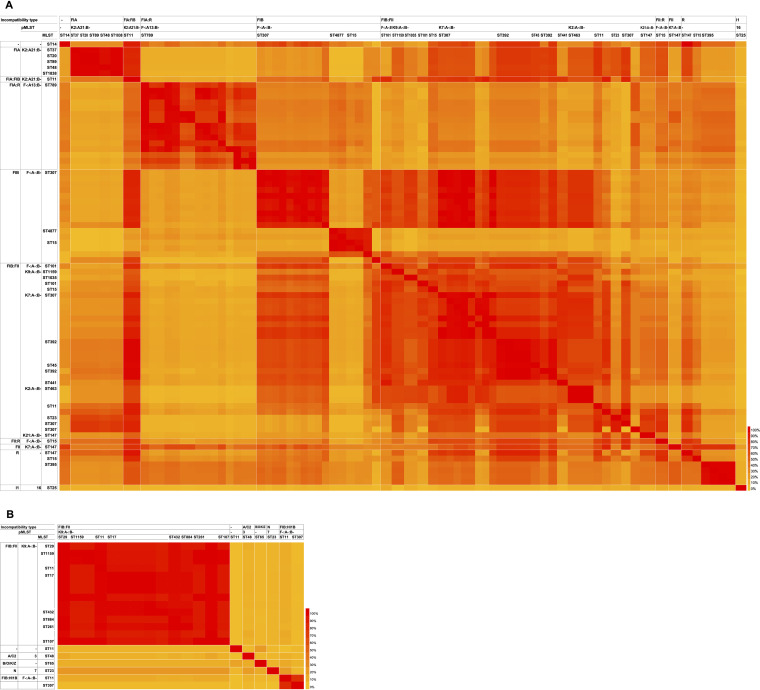
Heatmap of the pairwise coverage comparison of the group 1 (A) and group 9 (B) *bla*_CTX-M_ gene-carrying plasmids. The plasmids are ordered by incompatibility type, pMLST, and MLST of the bacterial host carrying the plasmid, and the plasmids are grouped based on the *bla*_CTX-M_ gene with white lines.

**(ii) Comparison between the *bla*_CTX-M_ gene-carrying plasmids.** Pairwise comparison of the plasmids for the percent coverage of the sequences having >99.5% nucleic acid identity presented high percent coverage association by incompatibility type and by plasmid MLST (pMLST) of the plasmid (Fig. 4). The relatively high percent coverage of intertypes of plasmids was observed through mosaicism. For instance, the FIA:FIB plasmid has higher coverage than the FIB and FIB:FII plasmids. Curiously high percent coverage grouping by the ST of the host bacteria was observed among the FIB and R plasmids, even in an incompatibility type and a pMLST. The prevalent *bla*_CTX-M-14_ gene-carrying FIB:FII plasmids were likely categorized into two groups: those harbored by ST29 and ST1159 and those harbored by others. The FIB:HI1B plasmids carrying either the *bla*_CTX-M-9_ or the *bla*_CTX-M-14_ genes had perfect coverage except for the region upstream from the *bla*_CTX-M_ gene. The plasmids belonging to the unique incompatibility types that lacked mosaicism, including I1, A/C, N, and B/O/K/Z, were incompatible with other types of plasmids presenting low percent coverage.

**(iii) Conjugation efficiency differed by the plasmid and the bacterial host.** The transfer efficiency of plasmids belonging to representative incompatibility types was determined by liquid mating using the ST307, ST375, and ST17 recipients. The FIA:RΩ*bla*_CTX-M-15_ plasmid transferred effectively to ST17 and one of the two ST307 isolates with transfer frequencies of 3.0 × 10^−5^ to 1.3 × 10^−4^ and 3.4 × 10^−6^ to 1.1 × 10^−5^, respectively, while the FIB:FIIΩ*bla*_CTX-M-14_ plasmid transferred to the other ST307 isolate with a frequency of 5.1 × 10^−6^ to 5.2 × 10^−5^ (Table 2). The results indicated the recipient preference of each plasmid. The differing efficiencies of plasmid transfer made a good correlation with the prevalence of CTX-M ESBLs in various STs.

**TABLE 2:** Conjugation efficiency^*a*^

Recipient	ST	Conjugation efficiency of donor:	
		C17KP0019 (ST789)/FIA:RΩ*bla*_CTX-M-15_	E16KP0235 (ST432)/FIB:FIIΩ*bla*_CTX-M-14_
B16KP0003	ST17	3.0 × 10^−5^ to 1.3 × 10^−4^	<1.0 × 10^−9^
F16KP0005	ST375	<1.0 × 10^−9^	<1.0 × 10^−9^
E16KP0152	ST307	<1.0 × 10^−9^	5.1 × 10^−6^ to 5.2 × 10^−5^

a The experiments were performed in duplicate and repeated at least three times.

### The *bla*_CTX-M-15_ gene integrated into chromosomes.

**(i) Bacterial clones carrying the *bla*_CTX-M_ gene in chromosomes.** The chromosomal location of the *bla*_CTX-M_ gene was observed for the *bla*_CTX-M-15_ gene in 29 K. pneumoniae isolates: 16 ST48, 8 ST307, and 1 each of ST14, ST15, ST101, ST392, and ST392-like, with one nucleotide difference at the *rpoB* allele (Fig. 2). Two copies of the *bla*_CTX-M-15_ gene were found on 17 chromosomes of all 16 ST48 isolates and 1 ST392-like isolate. Nine of the 29 K. pneumoniae isolates possessed a plasmid harboring the *bla*_CTX-M_ gene: the ST392-like isolate, one ST101 isolate, and two ST307 isolates possessed an FIB:FIIΩ*bla*_CTX-M-15_ plasmid; one ST15 isolate and two ST307 isolates had an FIIΩ*bla*_CTX-M-15_ plasmid; one ST48 isolate had an FIAΩ*bla*_CTX-M-15_ plasmid; and an ST48 isolate had an A/CΩ*bla*_CTX-M-14_ plasmid. A K. pneumoniae ST392 isolate had FIB and FII replication origins in the chromosome, and the plasmid-like 196,572-bp portion included the *bla*_CTX-M-15_ gene. The integration unit was bracketed by IS*26* copies in the same direction, and the possibility of any assembly error was rejected by direct repeats at both ends of the integration unit (Table S1). Intriguingly, the chromosomal sequences were similar to the 193,678-bp FIB:FII plasmid in the ST392-like isolate.

10.1128/mSystems.00459-20.1 Table S1 Chromosomal location of the *bla*_CTX-M-15_ gene and the characteristics of the integration. Download Table S1, DOCX file, 0.02 MB. Copyright © 2020 Yoon et al. 2020 Yoon et al. This content is distributed under the terms of the Creative Commons Attribution 4.0 International license.

### The integration units associated with the *bla*_CTX-M_ gene and the targeting locus.

**FIG 5:**
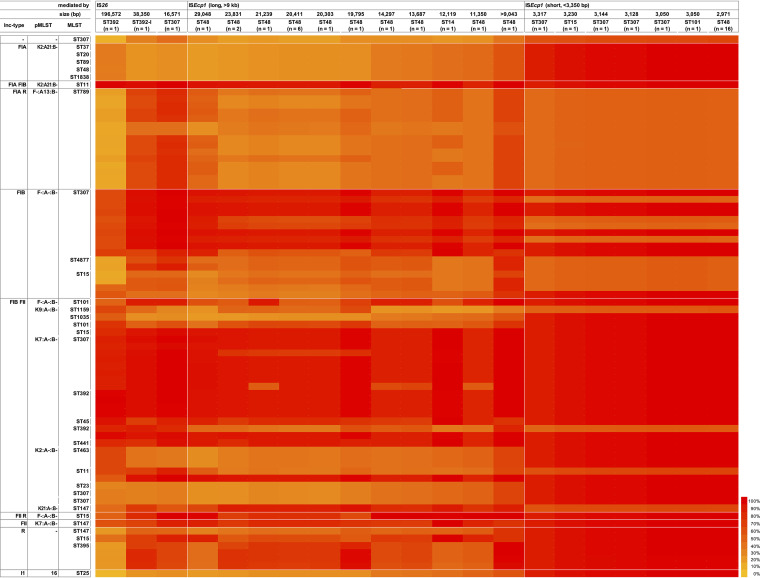
Heatmap of the pairwise coverage comparison between the group 1 *bla*_CTX-M_ gene-carrying plasmids and chromosomal integration units carrying the *bla*_CTX-M-15_ gene. The plasmids (raw) are ordered by incompatibility type, pMLST, and MLST of the bacterial host carrying the plasmid. The integration units (column) are ordered by the insertion sequences mediating the chromosomal integration and the size of the unit.

The integration was mediated either by IS*Ecp1* (*n* = 42) or by IS*26* (*n* = 3). Two integration events per chromosome, both mediated by IS*Ecp1*, were observed in all 16 ST48 isolates, and single integration by IS*Ecp1* was observed in seven of the eight ST307 isolates and one each of the ST101, ST14, and ST15 isolates. Chromosomal integration by IS*26* was found in one of the eight ST307 isolates and one each of the ST392 and ST392-like isolates. All of the targeted sites of integration were in the so-called core genome of K. pneumoniae, including the 16S rRNA and the coding sequences of OmpK35; except for the case found in ST15, the IS*Ecp1*-*bla*_CTX-M-15_-*orf477* unit transposon was integrated into a 42,544-bp prophage bracketed by a complete 64-bp *attL*-*attR* sequence. The sequential order and the integration of the prophage carrying the *bla*_CTX-M-15_ unit transposon or that of the *bla*_CTX-M-15_ unit transposon into the preintegrated prophage were debatable. The integrations by IS*Ecp1* generated 5-bp direct repeats and were 2,971 to 29,048 bp in length, and those by IS*26* were bracketed by 8-bp direct repeats and were 16,570 to 196,572 bp in length (Table S1). Comparison of the chromosomal integration units with plasmids for percent coverage of sequences having a nucleic acid identity of >99.5%, except for the IS*Ecp1*-*bla*_CTX-M-15_-*orf477* unit transposon, showed that the integration units were more covered by the FIB (F-:A-:B-) and FIB:FII (K7:A-:B-) plasmids hosted by ST307 (Fig. 5).

**FIG 6:**
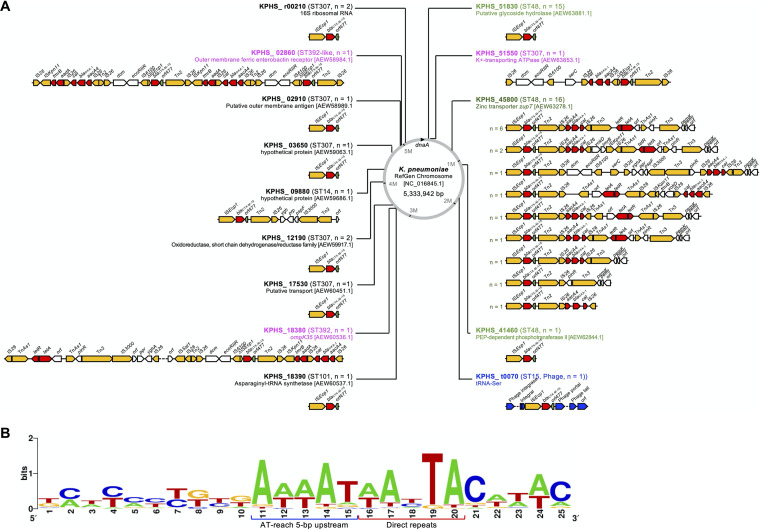
Chromosomal location of the *bla*_CTX-M-15_-associated integration units shown on the chromosome of the K. pneumoniae reference genome (NC_016845.1) and genomic contexts of the integration units (A) and the sequence logo of the chromosomal integration sites extracted from the regions 15 bp upstream and 5 bp downstream from the direct repeats (B) (21). (A) Locus tags are indicated with STs, and the number of isolates carrying the integration unit is indicated in parentheses. Open arrows indicate open reading frames, and the color codes indicate functions of each ORF: red, antimicrobial resistance determinants; green, *orf477* downstream from the *bla*_CTX-M_ gene; yellow, transposases of insertion sequences; blue, phage associated; white, others. (B) The sequences of direct repeats and AT-rich 5 bp upstream are represented.

Referring to the reference genome of K. pneumoniae (GenBank accession number NC_016845.1), integration occurred all over the chromosome, except between ca. 2 Mb and 3 Mb from the *dnaA* gene at the chromosomal replication origin (Fig. 6A). The chromosomal integrations of two *bla*_CTX-M-15_ copies were placed into two groups: (i) IS*26*-mediated tandem duplication of an 18,765-bp region, including the *bla*_CTX-M-15_ gene occurring in ST392, and (ii) sequential jumping of the IS*Ecp1*-*bla*_CTX-M-15_-*orf477* unit transposon from the primary integration unit found in ST48. For the second group, the primary integration identically targeted KPHS_45800 of the reference genome, and the ensuing integration was directed to KPHS_51830 or to KPHS_41460. The primary integration units interrupting KPHS_45800 had diverse structures, indicating independent events of each isolate. The second integration presumably had preferential sequences of the IS*Ecp1*-*bla*_CTX-M-15_-*orf477* unit transposon, and the sequence logo from the upstream and downstream sequences of the IS*Ecp1*-mediated integration sites presented a consensus of the 5-bp AT-rich sequences upstream from the direct repeats, while the sequences further upstream and downstream did not (Fig. 6B) (21).

### Promoter sequences of the *bla*_CTX-M_ gene.

Promoter sequences of the *bla*_CTX-M_ gene were provided from the upstream ISs (Fig. S3). All group 1 *bla*_CTX-M_ genes had an IS*Ecp1* copy 48 bp upstream from the gene except for the *bla*_CTX-M-3_ gene of the longest region, 124 bp. The 76-bp elongated region indicates that the *bla*_CTX-M-3_ gene-capturing event was independent from those of the other group 1 *bla*_CTX-M_ genes. In the case of the group 9 *bla*_CTX-M_ gene, the IS*Ecp1* copy was located 45 bp upstream from the *bla*_CTX-M-14_ gene, and IS*CR1* was found 115 bp upstream from the *bla*_CTX-M-9_ gene. The promoter sequences were identical if they were given by the upstream IS*Ecp1* copy.

10.1128/mSystems.00459-20.5 FIG S3 Aligned promoter sequences upstream from the *bla*_CTX-M_ genes identified in the K. pneumoniae blood strains. The −35 and −10 sequences, together with the transcriptional initiation site, are indicated with yellow shading. Sky blue and red shading indicates Shine Dalgarno and start sequences of the *bla*_CTX-M_ gene, respectively. The IS*Ecp1* sequences are indicated in red, and the inverted repeats are underlined. The sequence of IS*CR* is indicated in green. Download FIG S3, TIF file, 2.8 MB. Copyright © 2020 Yoon et al. 2020 Yoon et al. This content is distributed under the terms of the Creative Commons Attribution 4.0 International license.

## DISCUSSION

Third-generation cephalosporins are widely used in clinical settings to treat patients with K. pneumoniae bloodstream infections, and the increasing rate of cephalosporin resistance leads to increased use of carbapenems, encouraging the emerging carbapenem-resistant *Enterobacterales*. Currently, resistance to third-generation cephalosporins is mainly due to the acquisition of ESBL genes, and the genes mainly belong to the *bla*_CTX-M_ type. The dissemination of the gene has mostly been favored due to its location in incompatibility F-type plasmids, which are widely distributed in E. coli and K. pneumoniae (8) and are freely acquired and lost by the bacterial host as the antimicrobial environment changes. The chromosomal location of the resistance determinants has taken the situation to a new level of steady spread of resistance regardless of the habitat of the bacterial host, and particular attention needs to be paid to the widespread clinical isolates harboring the chromosomal *bla*_CTX-M_ gene.

The study was conceived through the observation of the resistance isolates belonging to some STs and the almost absolute prevalence of CTX-M-15 and CTX-M-14. Antimicrobial resistance in the dominant clone ST25 is indeed meager, and the ST307, ST789, ST11, and ST48 clones that make up no more than 4.5% of total K. pneumoniae blood isolates presented high rates of cefotaxime resistance between 78% and 100% (20). Even though an extra consideration for the presence of clonally related isolates is needed, the disproportional groups of the *bla*_CTX-M_ gene carried by each ST isolate were caused by, at least partially, the preferred plasmid type carrying the gene. It was likely that the particular clone has a preferential plasmid type and vice versa.

The base pair distance between the right end of the inverted repeat for IS*Ecp1* and the start codon of the *bla*_CTX-M_ gene could give a brief point of comparison for the international genetic contexts. The 48-bp distance for the group 1 *bla*_CTX-M_ genes found in the present study was frequently identified in other parts of the world, i.e., France, India, and China (22–24). However, the 124-bp distance for the *bla*_CTX-M-3_ gene and the 45-bp distance for the *bla*_CTX-M-14_ gene seemed unique to this study, and no identical sequence was found even from the nucleotide collection of the GenBank database.

The plasmids carrying the *bla*_CTX-M_ gene mostly belonged to the incompatibility F-type as a mosaic FIB:FII. Among the FIB:FII plasmids, those in the isolates belonging to the most prevalent CTX-M ESBL-producing ST307 seemed discrete in terms of its TA systems. The GNAT-related toxin, first identified in *Salmonella*, is known to inhibit translation and arrest further growth of the bacterial host (25). The rare incompatibility types were R, which is also famous as an antimicrobial resistance-associated plasmid, I1, carrying the group 1 *bla*_CTX-M_ type, and the N, A/C2, and B/O/K/Z types, harboring the group 9 *bla*_CTX-M_ gene. Compared to the previous reports (10, 13), the plasmid types became much more disproportionate. Among the 115 CTX-M ESBL-producing K. pneumoniae blood isolates in this study, more than a quarter of the isolates harbored the *bla*_CTX-M_ gene in their chromosomes. All of the chromosomal *bla*_CTX-M_ genes were subtype 15 and were harbored by K. pneumoniae hosts mostly belonging to ST48 and ST307, although the disproportionate distribution of *bla*_CTX-M_ gene-harboring clones was considered. ST307 and, less dominantly, ST48 are globally notorious K. pneumoniae carbapenemase (KPC)-producing clones, and both have been reported as KPC producers in South Korea (26).

More than a quarter of the ST307 isolates harbored the chromosomal *bla*_CTX-M-15_ gene. The gene was included in varied integration units in terms of length, which differed by the 3′ region of the unit transposon of IS*Ecp1*-*bla*_CTX-M-15_-*orf477*. The integration units found in ST307 targeted diverse loci in the chromosome, emphasizing the genome plasticity of the notorious clone. One of the isolates harbored the gene in an integration unit bracketed by a pair of IS*26* elements of a direction of the KHPS_51550 K^+^-transporting ATPase-coding sequence. Interestingly, the other seven ST307 isolates had one copy of IS*26* interrupting KHPS_51550, disclosing the IS*26*-anchored chromosomal integration of the *bla*_CTX-M-15_ gene-including segment in a plasmid. In this case, the IS*Ecp1* copy upstream from the *bla*_CTX-M-15_ gene was truncated, and the lost mobility was replaced with the IS*26* copy. Two other IS*26*-mediated integration cases in ST392 and ST392-like clones also included the truncated IS*Ecp1* copy upstream from the *bla*_CTX-M-15_ gene. Integration of the entire FIB:FII plasmid in the ST392 chromosome and that of the duplicated composite transposon in the ST392-like chromosome was bracketed by a pair of IS*26* elements in a particular direction. In the latter case, a supposed rolling-circle tandem amplification by IS*26* resulted in the double copy of the *bla*_CTX-M-15_ gene in a chromosome.

More than half of the isolates harboring the residential *bla*_CTX-M_ gene belonged to ST48, which had two copies of the gene at a distant locus in the chromosome. The ST48 isolates were clonally distinct, and the chromosomal integration events seemed to be independent. The integration was always mediated by IS*Ecp1*, and the integration hot spots were the zinc transporter *zupT* gene and the genes encoding phosphoenolpyruvate-dependent phosphotransferase II and putative glycoside hydrolase. The *zupT* gene was presumed to be a primary integration site for an IS*Ecp1* unit transposon from an R plasmid, because the unit size is longer than 9 kb and the genetic contexts included plasmid-associated components. At the other hot spot, the identical 2,971-bp unit transposon of IS*Ecp1*-*bla*_CTX-M-15_-*orf477* was identified as prevailing in the second integration, probably from the primary integration unit. In one exceptional ST48 case, secondary integration was observed at the other gene as a 3′-terminal truncated form of the unit transposon of IS*Ecp1*-*bla*_CTX-M-15_-*orf477*.

The targeted integration locus had a peculiar consensus AT-rich sequence 5 bp upstream from the direct repeats. This integration site was preferred not only by the major clones but also by the minor ST101, ST14, and ST15 clones. An ST15 isolate was infected by a prophage, and the chromosomal integration of the unit transposon IS*Ecp1*-*bla*_CTX-M-15_-*orf477* targeted the AT-rich region within the prophage. Based on the rareness of integration mediated by IS*26*, complex contexts of the IS*26*-associated integration unit and identified truncated IS*Ecp1* copy made it possible to form a reasonable hypothesis: IS*26* would be the second-best choice, following IS*Ecp1*, for the chromosomal integration of the *bla*_CTX-M_ gene.

The cefotaxime-resistant K. pneumoniae clones of this study were particularized because of the fastidiousness for plasmids to acquire the *bla*_CTX-M_ gene and of the chromosomal accumulation of the *bla*_CTX-M_ gene. The fit clones in clinical settings may have performed a consequent dissemination through acquired resistance, during which the present population of K. pneumoniae blood isolates was likely being made. The diverse genetic contexts bracketing the *bla*_CTX-M-15_ gene, chromosomal locus of integration, and the hospitals from which the isolates were recovered provided enough evidence to make an assumption. Thus far, to the best of our knowledge, nationwide dissemination of K. pneumoniae clones with the residential *bla*_CTX-M_ gene has never been reported, and we consider it important to keep a close watch on its status.

This study showed an evolutionary path for antimicrobial resistance in clinical isolates to sustain their life while surrounded by an abundance of antimicrobials. The evolutionary strategy could be summed up in two parts, securing diversity and ensuring propagation. For diversification, different types of plasmids were equipped for enough trials of the bacterial host, and various accessible mobile genetic elements were used to acquire the gene. Such a plan is important for the bacterial host to avoid being at a standstill. To ensure stable propagation, the antimicrobial resistance determinant was appointed as a residential gene in the chromosome. The bacterial host then could better deal with encountering the life-threatening antimicrobials.

## MATERIALS AND METHODS

### Isolates used in the study.

Among the 134 isolates collected from the cohort study, a total of 115 isolates harboring the *bla*_CTX-M_ genes were recoverable in good shape, and those 115 isolates were used for the study.

### Whole-genome sequencing.

From the 115 K. pneumoniae isolates, genomic DNA was extracted with the GenElute bacterial genomic DNA kit (Sigma-Aldrich, St. Louis, MO). The entire genomes were sequenced using both Illumina and Nanopore technologies. Libraries were prepared for Illumina using both the Swift 2S Turbo DNA library kit (Swift Biosciences, Ann Arbor, MI) and Swift 2S Turbo combinatorial dual indexing primer kit (Swift Biosciences) and for Nanopore using the ligation sequencing kit (Oxford Nanopore, Oxford, UK). Reads were assembled using Spades (ver. 3.11.1) (27). Annotation of the complete sequences was carried out using prokka 1.13.7 (https://github.com/tseemann/prokka) (28).

### Phylogenetic analysis.

A total of 16 housekeeping proteins of the 115 K. pneumoniae core genomes were used to produce a multiple alignment with muscle v3.8 (29). The phylogeny was analyzed using PhyML v3.0 with the Whelan and Goldman matrix, and a gamma correction was made. To ensure the robustness of the topology, 100 bootstraps were calculated for the concatenated sequences. To root the phylogenetic tree, the genome of Klebsiella oxytoca CAV1374 (NZ_CP011636.1) was used.

### *In silico* molecular epidemiology study using the whole genome.

For multilocus sequence typing (MLST), allele numbers of seven housekeeping genes, *gapA*, *infB*, *mdh*, *pgi*, *phoE*, *rpoB*, and *tonB*, of K. pneumoniae were extracted by using MLST 2.0 (https://cge.cbs.dtu.dk/services/MLST/), and the corresponding ST was obtained through the procedures of Diancourt et al. (30). For six dominant STs, cgMLST, which is implemented in BIGSdb-Kl (https://bigsdb.pasteur.fr), was further carried out using a total of 2,537 loci (31). The relatedness of each isolate was inferred through constructing minimum spanning trees using PHYLOViZ (32). The identification of resistance determinants was assessed by using ResFinder (https://cge.cbs.dtu.dk//services/ResFinder/) (33). The incompatibility type of the *bla*_CTX-M_ gene-harboring plasmid and the plasmid MLST (pMLST) were determined by plasmid finder (https://cge.cbs.dtu.dk//services/PlasmidFinder/) and pMLST (https://cge.cbs.dtu.dk//services/pMLST/), respectively (34). Type II toxin/antitoxin systems were searched against the database of TADB 2.0 (35), and subtyping of SHV and CTX-M was conducted using a laboratory-made database.

### Plasmid transfer by bacterial conjugation.

For bacterial conjugation, spontaneous mutants resistant to both nalidixic acid and sodium azide were generated from drug-susceptible K. pneumoniae clinical isolates B16KP0003 of ST17, F16KP0005 of ST375, and E16KP0152 and C16KP0023 of ST307, which are devoid of any obvious plasmid by electrophoresis, for recipients. K. pneumoniae ST789 C17KP0019/FIA:RΩ*bla*_CTX-M-15_ and ST432 E16KP0235/FIB:FIIΩ*bla*_CTX-M-14_ were selected as donors. Equal amounts of exponential cultures of the donor and recipient isolates were mixed, incubated in Mueller-Hinton broth devoid of any drug for 12 h, and spread on brain heart infusion agar (Difco Laboratories) containing nalidixic acid (30 mg/liter), sodium azide (100 mg/liter), and cefotaxime (10 mg/liter). Each colony was confirmed by PCR, and the plasmid transfer frequency was calculated as the number of transconjugants per donor. The experiments were performed in duplicate and repeated at least three times.

### Data availability.

The genomes of the 115 K. pneumoniae isolates were deposited in the GenBank nucleotide database under accession numbers CP052136–CP052744 (see Table S2 in the supplemental material) and under BioProject PRJNA625837.

10.1128/mSystems.00459-20.2 Table S2 GenBank accession numbers of the genome of K. pneumoniae strains used in the study. Download Table S2, DOCX file, 0.03 MB. Copyright © 2020 Yoon et al. 2020 Yoon et al. This content is distributed under the terms of the Creative Commons Attribution 4.0 International license.
