# Supplementary figures and images for: Beneficial Chromosomal Integration of the Genes for CTX-M Extended-Spectrum β-Lactamase in Klebsiella pneumoniae for Stable Propagation
Source: mSystems. 2020 Sep 29;5(5):e00459-20. doi: 10.1128/mSystems.00459-20 (PMC7527135; doi:10.1128/mSystems.00459-20)

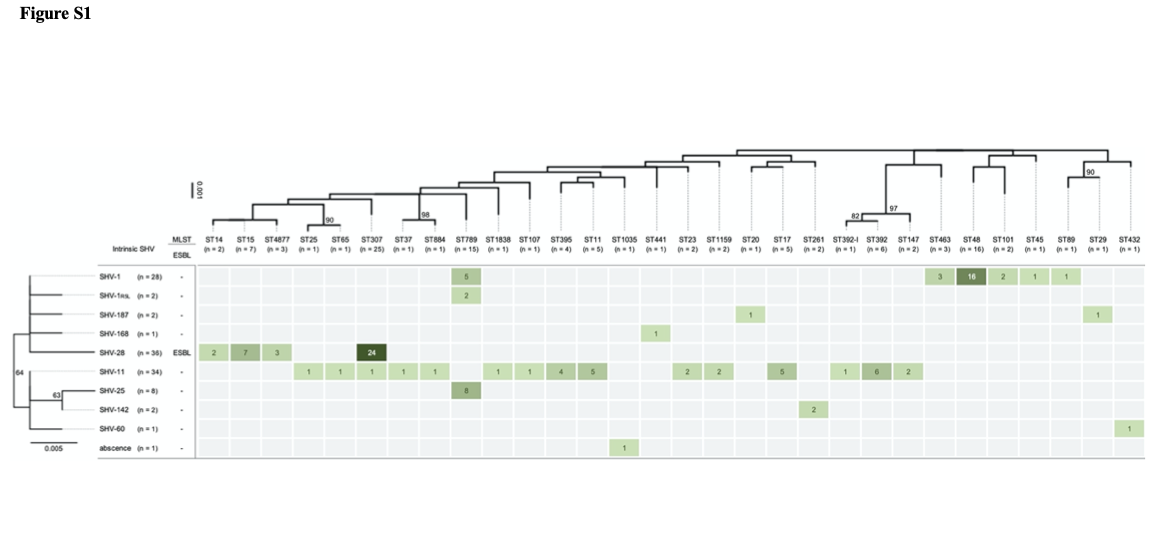

Supplement: FIG S1 [file mSystems.00459-20-sf001.tif]

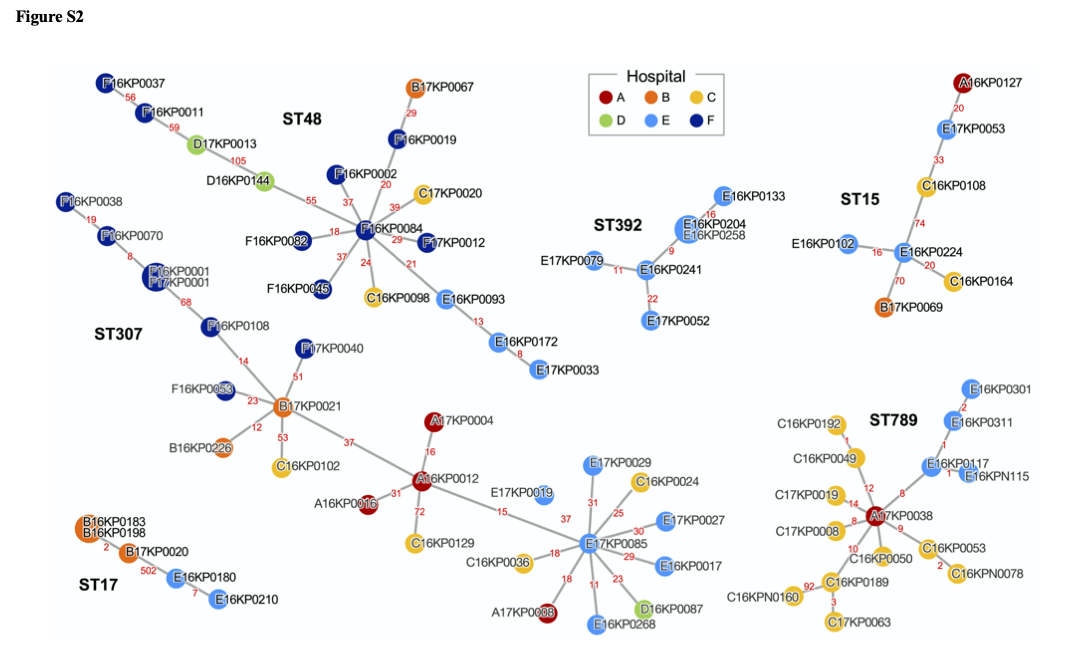

Supplement: FIG S2 [file mSystems.00459-20-sf002.tif]

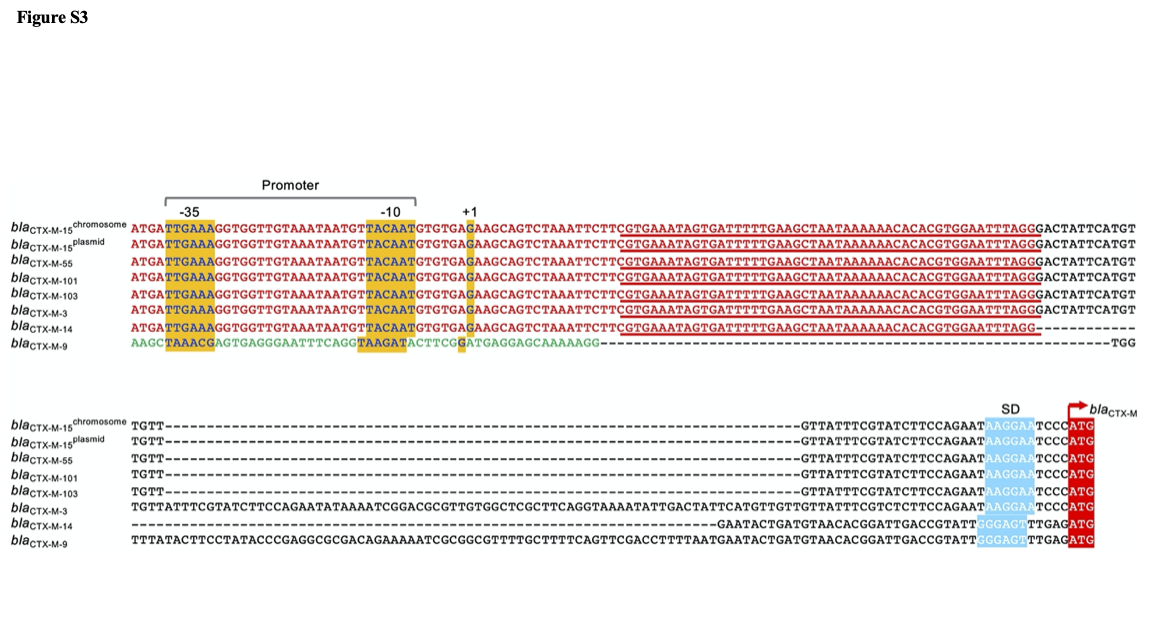

Supplement: FIG S3 [file mSystems.00459-20-sf003.tif]
