# Supplementary material for: Beneficial Chromosomal Integration of the Genes for CTX-M Extended-Spectrum β-Lactamase in Klebsiella pneumoniae for Stable Propagation
Source: mSystems. 2020 Sep 29;5(5):e00459-20. doi: 10.1128/mSystems.00459-20 (PMC7527135; doi:10.1128/mSystems.00459-20)
Supplement: Table S1 [file mSystems.00459-20-st001.docx]

**Table S1. Chromosomal location of the *bla*_CTX-M-15_ gene and the characteristics of the integration.**

| Strain | Site | MLST | Intrinsic  SHV | #1 | | | | | #2 | | | | |
| --- | --- | --- | --- | --- | --- | --- | --- | --- | --- | --- | --- | --- | --- |
|  |  |  |  | KPHS_ | Size | mediated by | Integration site | DR | KPHS_ | Size | mediated by | Integration site | DR |
| E16KP0212 | E | ST392-l | SHV-11 | 2860 | 38,350 | IS*26* | CGCAGGTCAGCGCCTTCC | GTCAGCGC | - | - | - | - | - |
| E16KP0258 | E | ST392 | SHV-11 | 18380 | 196,572 | IS*26* | ATATAAAAAATATTAATG | AAAAATAT | - | - | - | - | - |
| F16KP0096 | F | ST101 | SHV-1 | 18390 | 3,050 | IS*Ecp1* | ATAAAAAATATTAAT | AAATA | - | - | - | - | - |
| F16KP0045 | F | ST48 | SHV-1 | 45800 | 19,795 | IS*Ecp1* | AAAATAATTACATAC | AATTA | 51830 | 2,971 | IS*Ecp1* | ATTATTACTACTATC | TACTA |
| C17KP0020 | C | ST48 | SHV-1 | 45800 | 29,048 | IS*Ecp1* | AAAATAATTACATAC | AATTA | 41460 | >2,848 | IS*Ecp1* | GTTACAGATAAATAA | AGATA* |
| F17KP0012 | F | ST48 | SHV-1 | 45800 | 11,350 | IS*Ecp1* | AAAATAATTACATAC | AATTA | 51830 | 2,971 | IS*Ecp1* | ATTATTACTACTATC | TACTA |
| F16KP0084 | F | ST48 | SHV-1 | 45800 | 21,239 | IS*Ecp1* | AAAATAATTACATAC | AATTA | 51830 | 2,971 | IS*Ecp1* | ATTATTACTACTATC | TACTA |
| F16KP0082 | F | ST48 | SHV-1 | 45800 | 13,687 | IS*Ecp1* | AAAATAATTACATAC | AATTA | 51830 | 2,971 | IS*Ecp1* | ATTATTACTACTATC | TACTA |
| F16KP0037 | F | ST48 | SHV-1 | 45800 | 14,297 | IS*Ecp1* | AAAATAATTACATAC | AATTA | 51830 | 2,971 | IS*Ecp1* | ATTATTACTACTATC | TACTA |
| F16KP0019 | F | ST48 | SHV-1 | 45800 | >9,043 | IS*Ecp1* | AAAATAATTACATAC | AATTA* | 51830 | 2,971 | IS*Ecp1* | ATTATTACTACTATC | TACTA |
| F16KP0011 | F | ST48 | SHV-1 | 45800 | 23,831 | IS*Ecp1* | AAAATAATTACATAC | AATTA | 51830 | 2,971 | IS*Ecp1* | ATTATTACTACTATC | TACTA |
| F16KP0002 | F | ST48 | SHV-1 | 45800 | 20,411 | IS*Ecp1* | AAAATAATTACATAC | AATTA | 51830 | 2,971 | IS*Ecp1* | ATTATTACTACTATC | TACTA |
| E17KP0033 | E | ST48 | SHV-1 | 45800 | 20,411 | IS*Ecp1* | AAAATAATTACATAC | AATTA | 51830 | 2,971 | IS*Ecp1* | ATTATTACTACTATC | TACTA |
| E16KP0172 | E | ST48 | SHV-1 | 45800 | 23,831 | IS*Ecp1* | AAAATAATTACATAC | AATTA | 51830 | 2,971 | IS*Ecp1* | ATTATTACTACTATC | TACTA |
| E16KP0093 | E | ST48 | SHV-1 | 45800 | 20,411 | IS*Ecp1* | AAAATAATTACATAC | AATTA | 51830 | 2,971 | IS*Ecp1* | ATTATTACTACTATC | TACTA |
| D17KP0013 | D | ST48 | SHV-1 | 45800 | 20,411 | IS*Ecp1* | AAAATAATTACATAC | AATTA | 51830 | 2,971 | IS*Ecp1* | ATTATTACTACTATC | TACTA |
| D16KP0144 | D | ST48 | SHV-1 | 45800 | 20,411 | IS*Ecp1* | AAAATAATTACATAC | AATTA | 51830 | 2,971 | IS*Ecp1* | ATTATTACTACTATC | TACTA |
| B17KP0067 | B | ST48 | SHV-1 | 45800 | 20,411 | IS*Ecp1* | AAAATAATTACATAC | AATTA | 51830 | 2,971 | IS*Ecp1* | ATTATTACTACTATC | TACTA |
| C16KP0098 | C | ST48 | SHV-1 | 45800 | 20,303 | IS*Ecp1* | AAAATAATTACATAC | AATTA | 51830 | 2,971 | IS*Ecp1* | ATTATTACTACTATC | TACTA |
| E16KP0035 | E | ST14 | SHV-28 | 9880 | 12,119 | IS*Ecp1* | TAATGCTGTTGATAA | CTGTT | - | - | - | - | - |
| C16KP0108 | C | ST15 | SHV-28 | t00700** | 3,230 | IS*Ecp1* | GTAATTTTTACCTAG | TTTTA | - | - | - | - | - |
| E17KP0027 | E | ST307 | SHV-28 | 12190 | 3,128 | IS*Ecp1* | ACAGAATATTGTCAA | ATATT | - | - | - | - | - |
| E16KP0017 | E | ST307 | SHV-28 | 12190 | 3,144 | IS*Ecp1* | ACAGAATATTGTCAA | ATATT | - | - | - | - | - |
| C16KP0129 | C | ST307 | SHV-28 | 3650 | 3,050 | IS*Ecp1* | TATTCAAATACGTTA | AAATA | - | - | - | - | - |
| C16KP0024 | C | ST307 | SHV-28 | r00210 | 3,317 | IS*Ecp1* | AATTACTGGGCGCAA | CTGGG | - | - | - | - | - |
| B17KP0021 | B | ST307 | SHV-28 | 2910 | 2,971 | IS*Ecp1* | AAAAGTACGATGGTG | TACGA | - | - | - | - | - |
| B16KP0226 | B | ST307 | SHV-28 | 2910 | 2,971 | IS*Ecp1* | AAAAGTACGATGGTG | TACGA | - | - | - | - | - |
| A16KP0012 | A | ST307 | SHV-28 | 51550 | 16,571 | IS*26* | AACCAGCCTGCTGAGCAG | GCCTGCTG | - | - | - | - | - |
| A16KP0016 | A | ST307 | SHV-28 | 17530 | 2,971 | IS*Ecp1* | ATCAGTCATAAGTAA | TCATA | - | - | - | - | - |

*Right copy of the direct repeat was absent due to genetic recombination.

**The unit was integrated into a prophage at the locus.
