# Supplementary material for: Beneficial Chromosomal Integration of the Genes for CTX-M Extended-Spectrum β-Lactamase in Klebsiella pneumoniae for Stable Propagation
Source: mSystems. 2020 Sep 29;5(5):e00459-20. doi: 10.1128/mSystems.00459-20 (PMC7527135; doi:10.1128/mSystems.00459-20)
Supplement: Table S2 [file mSystems.00459-20-st002.docx]

**Table S2. GenBank accession of the genome of *K. pneumoniae* strains used in the study.**

| *K. pneumoniae* | Number of contigs | GenBank accession | | | |
| --- | --- | --- | --- | --- | --- |
|  |  | Chromosome | Plasmids | | |
| A16KP0012 | 1 | CP052573 |  | - |  |
| A16KP0016 | 2 | CP052571 |  |  | CP052572 |
| A16KP0119 | 2 | CP052569 |  |  | CP052570 |
| A16KP0127 | 4 | CP052565 | CP052566 | to | CP052568 |
| A16KP0135 | 3 | CP052562 | CP052563 | and | CP052564 |
| A17KP0004 | 2 | CP052560 |  |  | CP052561 |
| A17KP0008 | 3 | CP052557 | CP052558 | and | CP052559 |
| A17KP0038 | 6 | CP052551 | CP052552 | to | CP052556 |
| B16KP0048 | 11 | CP052716 | CP052717 | to | CP052726 |
| B16KP0078 | 10 | CP052706 | CP052707 | to | CP052715 |
| B16KP0089 | 5 | CP052546 | CP052547 | to | CP052550 |
| B16KP0102 | 2 | CP052544 |  |  | CP052545 |
| B16KP0141 | 7 | CP052537 | CP052538 | to | CP052543 |
| B16KP0157 | 4 | CP052533 | CP052534 | to | CP052536 |
| B16KP0177 | 9 | CP052524 | CP052525 | to | CP052532 |
| B16KP0183 | 2 | CP052522 |  |  | CP052523 |
| B16KP0198 | 2 | CP052520 |  |  | CP052521 |
| B16KP0202 | 10 | CP052510 | CP052511 | to | CP052519 |
| B16KP0226 | 5 | CP052505 | CP052506 | to | CP052509 |
| B17KP0020 | 2 | CP052503 |  |  | CP052504 |
| B17KP0021 | 5 | CP052498 | CP052499 | to | CP052502 |
| B17KP0067 | 4 | CP052494 | CP052495 | to | CP052497 |
| B17KP0069 | 4 | CP052490 | CP052491 | to | CP052493 |
| C16KP0024 | 3 | CP052487 | CP052488 | and | CP052489 |
| C16KP0036 | 3 | CP052484 | CP052485 | and | CP052486 |
| C16KP0049 | 8 | CP052698 | CP052699 | to | CP052705 |
| C16KP0050 | 8 | CP052476 | CP052477 | to | CP052483 |
| C16KP0053 | 8 | CP052468 | CP052469 | to | CP052475 |
| C16KP0065 | 17 | CP052451 | CP052452 | to | CP052467 |
| C16KP0077 | 2 | CP052449 |  |  | CP052450 |
| C16KP0078 | 11 | CP052687 | CP052688 | to | CP052697 |
| C16KP0098 | 6 | CP052443 | CP052444 | to | CP052448 |
| C16KP0102 | 3 | CP052440 | CP052441 | and | CP052442 |
| C16KP0108 | 6 | CP052434 | CP052435 | to | CP052439 |
| C16KP0122 | 3 | CP052431 | CP052432 | and | CP052433 |
| C16KP0129 | 4 | CP052427 | CP052428 | to | CP052430 |
| C16KP0160 | 9 | CP052736 | CP052737 | to | CP052744 |
| C16KP0164 | 5 | CP052422 | CP052423 | to | CP052426 |
| C16KP0189 | 8 | CP052414 | CP052415 | to | CP052421 |
| C16KP0192 | 8 | CP052679 | CP052680 | to | CP052686 |
| C17KP0008 | 7 | CP052407 | CP052408 | to | CP052413 |
| C17KP0019 | 8 | CP052671 | CP052672 | to | CP052678 |
| C17KP0020 | 3 | CP052404 | CP052405 | and | CP052406 |
| C17KP0033 | 9 | CP052662 | CP052663 | to | CP052670 |
| C17KP0039 | 5 | CP052399 | CP052400 | to | CP052403 |
| C17KP0040 | 7 | CP052392 | CP052393 | to | CP052398 |
| C17KP0052 | 4 | CP052388 | CP052389 | to | CP052391 |
| C17KP0055 | 2 | CP052386 |  |  | CP052387 |
| C17KP0063 | 8 | CP052654 | CP052655 | to | CP052661 |
| D16KP0008 | 5 | CP052381 | CP052382 | to | CP052385 |
| D16KP0017 | 2 | CP052379 |  |  | CP052380 |
| D16KP0025 | 4 | CP052375 | CP052376 | to | CP052378 |
| D16KP0042 | 3 | CP052372 | CP052373 | and | CP052374 |
| D16KP0087 | 3 | CP052369 | CP052370 | and | CP052371 |
| D16KP0109 | 4 | CP052365 | CP052366 | to | CP052368 |
| D16KP0122 | 3 | CP052362 | CP052363 | and | CP052364 |
| D16KP0144 | 6 | CP052356 | CP052357 | to | CP052361 |
| D16KP0146 | 5 | CP052351 | CP052352 | to | CP052355 |
| D17KP0013 | 7 | CP052344 | CP052345 | to | CP052350 |
| D17KP0018 | 8 | CP052336 | CP052337 | to | CP052343 |
| D17KP0022 | 4 | CP052332 | CP052333 | to | CP052335 |
| D17KP0032 | 4 | CP052328 | CP052329 | to | CP052331 |
| E16KP0017 | 2 | CP052326 |  |  | CP052327 |
| E16KP0032 | 2 | CP052324 |  |  | CP052325 |
| E16KP0035 | 4 | CP052320 | CP052321 | to | CP052323 |
| E16KP0093 | 5 | CP052315 | CP052316 | to | CP052319 |
| E16KP0102 | 6 | CP052309 | CP052310 | to | CP052314 |
| E16KP0115 | 19 | CP052635 | CP052636 | to | CP052653 |
| E16KP0117 | 8 | CP052627 | CP052628 | to | CP052634 |
| E16KP0133 | 3 | CP052306 | CP052307 | and | CP052308 |
| E16KP0172 | 3 | CP052303 | CP052304 | and | CP052305 |
| E16KP0180 | 2 | CP052301 |  |  | CP052302 |
| E16KP0204 | 4 | CP052297 | CP052298 | to | CP052300 |
| E16KP0210 | 2 | CP052295 |  |  | CP052296 |
| E16KP0212 | 3 | CP052292 | CP052293 | and | CP052294 |
| E16KP0218 | 6 | CP052286 | CP052287 | to | CP052291 |
| E16KP0224 | 6 | CP052280 | CP052281 | to | CP052285 |
| E16KP0235 | 2 | CP052278 |  |  | CP052279 |
| E16KP0241 | 3 | CP052275 | CP052276 | and | CP052277 |
| E16KP0258 | 3 | CP052272 | CP052273 | and | CP052274 |
| E16KP0268 | 4 | CP052268 | CP052269 | to | CP052271 |
| E16KP0287 | 3 | CP052265 | CP052266 | and | CP052267 |
| E16KP0288 | 3 | CP052262 | CP052263 | and | CP052264 |
| E16KP0290 | 4 | CP052258 | CP052259 | to | CP052261 |
| E16KP0301 | 14 | CP052244 | CP052245 | to | CP052257 |
| E16KP0311 | 8 | CP052619 | CP052620 | to | CP052626 |
| E17KP0019 | 3 | CP052241 | CP052242 | and | CP052243 |
| E17KP0027 | 2 | CP052239 |  |  | CP052240 |
| E17KP0029 | 4 | CP052235 | CP052236 | to | CP052238 |
| E17KP0033 | 4 | CP052231 | CP052232 | to | CP052234 |
| E17KP0052 | 7 | CP052224 | CP052225 | to | CP052230 |
| E17KP0053 | 7 | CP052217 | CP052218 | to | CP052223 |
| E17KP0079 | 4 | CP052213 | CP052214 | to | CP052216 |
| E17KP0085 | 6 | CP052207 | CP052208 | to | CP052212 |
| F16KP0001 | 11 | CP052608 | CP052609 | to | CP052618 |
| F16KP0002 | 4 | CP052203 | CP052204 | to | CP052206 |
| F16KP0011 | 6 | CP052197 | CP052198 | to | CP052202 |
| F16KP0014 | 5 | CP052192 | CP052193 | to | CP052196 |
| F16KP0019 | 3 | CP052189 | CP052190 | and | CP052191 |
| F16KP0037 | 8 | CP052181 | CP052182 | to | CP052188 |
| F16KP0038 | 12 | CP052596 | CP052597 | to | CP052607 |
| F16KP0045 | 4 | CP052177 | CP052178 | to | CP052180 |
| F16KP0050 | 2 | CP052175 |  |  | CP052176 |
| F16KP0053 | 9 | CP052727 | CP052728 | to | CP052735 |
| F16KP0064 | 3 | CP052172 | CP052173 | and | CP052174 |
| F16KP0070 | 11 | CP052585 | CP052586 | to | CP052595 |
| F16KP0075 | 5 | CP052167 | CP052168 | to | CP052171 |
| F16KP0082 | 5 | CP052162 | CP052163 | to | CP052166 |
| F16KP0084 | 4 | CP052158 | CP052159 | to | CP052161 |
| F16KP0096 | 8 | CP052150 | CP052151 | to | CP052157 |
| F16KP0108 | 3 | CP052147 | CP052148 | and | CP052149 |
| F17KP0001 | 11 | CP052574 | CP052575 | to | CP052584 |
| F17KP0012 | 4 | CP052143 | CP052144 | to | CP052146 |
| F17KP0040 | 5 | CP052138 | CP052139 | to | CP052142 |
| F17KP0054 | 2 | CP052136 |  |  | CP052137 |
